# The involvement of Purkinje cells in progressive myoclonic epilepsy: Focus on neuronal ceroid lipofuscinosis

**DOI:** 10.1016/j.nbd.2023.106258

**Published:** 2023-09

**Authors:** Sara Bernardi, Federica Gemignani, Maria Marchese

**Affiliations:** a Department Neurobiology and Molecular Medicine, IRCCS Fondazione Stella Maris, 56128 Pisa, Italy; b Department of Biology, University of Pisa, Pisa, Italy

**Keywords:** Purkinje cells, Progressive Myoclonic Epilepsy, seizures, Neuronal Ceroid Lipofuscinosis, neurodegenerative diseases

## Abstract

The progressive myoclonic epilepsies (PMEs) are a group of rare neurodegenerative diseases characterized by myoclonus, epileptic seizures, and progressive neurological deterioration with cerebellar involvement. They include storage diseases like Gaucher disease, Lafora disease, and forms of neuronal ceroid lipofuscinosis (NCL). To date, 13 NCLs have been reported (CLN1-CLN8, CLN10-CLN14), associated with mutations in different genes. These forms, which affect both children and adults, are characterized by seizures, cognitive and motor impairments, and in most cases visual loss. In NCLs, as in other PMEs, central nervous system (CNS) neurodegeneration is widespread and involves different subpopulations of neurons. One of the most affected regions is the cerebellar cortex, where motor and non-motor information is processed and transmitted to deep cerebellar nuclei through the axons of Purkinje cells (PCs). PCs, being GABAergic, have an inhibitory effect on their target neurons, and provide the only inhibitory output of the cerebellum. Degeneration of PCs has been linked to motor impairments and epileptic seizures. Seizures occur when some insult upsets the normal balance in the CNS between excitatory and inhibitory impulses, causing hyperexcitability. Here we review the role of PCs in epilepsy onset and progression following their PME-related loss. In particular, we focus on the involvement of PCs in seizure phenotype in NCLs, highlighting findings from case reports and studies of animal models in which epilepsy can be linked to PC loss.

## Highlights

• Purkinje cell degeneration is described in many neurodegenerative diseases with cognitive and motor impairment.
• Neuronal degeneration of GABAergic Purkinje cells (PCs) upsets the balance between inhibitory/excitatory signals.
• Seizures in progressive myoclonic epilepsy (PME) disorders are invalidating and mostly intractable.
• PME disorders include neuronal ceroid lipofuscinosis, in which loss of PCs is linked to seizure onset and motor phenotype.

## 1 Introduction

The cerebellum has been implicated in numerous neurological diseases characterised, in particular, by degeneration and morphological alteration of its Purkinje cells (PCs) (Cook et al., 2021).

PCs are pear-shaped cells with large somas that form a layer in the cerebellar cortex. They have a large dendritic tree enabling them to process all input from parallel fibres coming from around 200,000 granular cells and from climbing fibres originating from the inferior olivary nuclei (D'Angelo et al., 2011). Being responsible for integrating signals in the cerebellar cortex and then transmitting them externally, these neurons are the most important cells in the cerebellum (Cook et al., 2021). The cerebellum plays a key role in controlling locomotion and maintaining motor coordination, as well as in non-motor functions including cognitive processes and executive control (Koziol et al., 2014). PCs are known primarily as the encoding centre for motor behaviour and have indeed been found to be rhythmically active during locomotion (Chang et al., 2021).

The fact that a decrease in the number of PCs is commonly seen in many neurodegenerative disorders with motor dysfunction and dementia, such as Alzheimer’s disease (AD), Parkinson’s disease (PD), Huntington’s disease (HD), ataxias and progressive myoclonic epilepsy (PME) (Huang and Verbeek, 2019; Erekat, 2022), suggests that these cells are particularly vulnerable to neurodegeneration (Cook et al., 2021). However, it remains unclear why PCs are subject to degeneration and loss (Streng and Krook-Magnuson, 2021). The mechanisms underlying the progressive death of PCs differ between neurodegenerative diseases. Three processes — autophagy, apoptosis, and necrosis — have been linked to PC death (Erekat, 2022), and they are all associated with endoplasmic reticulum, mitochondrial and lysosomal impairment, three of the most common neurodegenerative disease pathways. Moreover, PCs are GABAergic neurons that provide inhibitory output to the deep cerebellar nuclei, loss of which has been observed in animal models and patients with epilepsy (Dam et al., 1984; Rajjoub et al., 1976), suggesting an association between cerebellar disinhibition and epileptogenesis (Ming et al., 2021).

The term PME refers to a large group of rare neurodegenerative diseases that share some main features with more common diseases, such as PD, AD, Down’s syndrome and HD (Yan et al., 2013; D’Orsi and Specchio, 2014; Thakor et al., 2021). Indeed, PME patients show myoclonus, ataxia, seizures, cognitive impairments such as dementia, and progressive neurological deterioration. This group of diseases includes storage diseases (neuronal ceroid lipofuscinosis, Gaucher disease and Lafora disease), mitochondrial encephalopathy (myoclonic epilepsy with ragged red fibres, MERRF), and deep brain degeneration (dentato-rubro-pallidoluysian atrophy, DRPLA) (Zupanc and Legros, 2004). To date, there are no efficient treatments for the different form of PME, which thus lead to rapid motor and cognitive decline. Different PMEs are found among the various neurological diseases characterized by seizures and PC loss.

The neuronal ceroid lipofuscinoses (NCLs) are a subgroup of PMEs characterized by accumulation of autofluorescent material in both neuronal and non-neuronal cells (Mole et al., 2005). They, too, are characterised by visual impairment, balance issues, myoclonus, epilepsy and motor and cognitive decline (Zupanc and Legros, 2004; Sinha et al., 2007; Bennett and Rakheja, 2013; Nita et al., 2016; Specchio et al., 2021). To date, 13 NCLs caused by mutations in *CLN* genes (*CLN1*–*CLN8*, *CLN10*–*CLN14*) have been reported (Schulz et al., 2013). NCLs can be grouped by age at onset into infantile (INCL), late-infantile (LINCL), juvenile (JNCL) and adult (ANCL) forms. Treatments are only palliative, and the disease progresses rapidly leading to premature death (Kohlschütter et al., 2019). PC degeneration has been described in many forms of NCL, and a correlation has been reported between cell impairment and disease progression (Hachiya et al., 2006).

In view of the role of PCs in motor coordination and their involvement in neurodegenerative diseases, this review aims to explore literature evidence on the role of PCs in seizures, particularly PMEs; a further aim is to examine in depth how loss of PCs contributes to the epileptic phenotype in NCLs, both at onset and during progression of the disease, in order to correlate pathological features with impairment of these cells.

## 2 Materials and methods

**Fig. 1:**
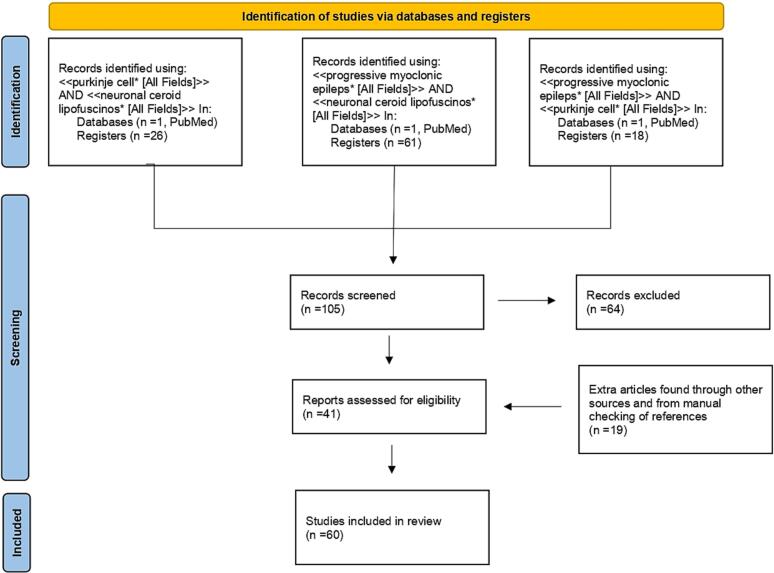
PRISMA 2020 flow diagram of the literature search process.

The PubMed database was queried using the following three search strings: <<purkinje cell*>> AND <<neuronal ceroid lipofuscinos* [All Fields]>>, <<progressive myoclonic epileps* [All Fields]>> AND <<neuronal ceroid lipofuscinos* [All Fields]>>, <<progressive myoclonic epileps* [All Fields]>> AND <<purkinje cell* [All Fields]>>. Articles retrieved had to be full-text articles written in English, and they had to have been published by October 15, 2022. Application of the three strings yielded 26, 61 and 18 publications, respectively. We then performed a manual search of the references listed in publications found to discuss the role of PCs in the onset and progression of PMEs, in particular NCLs. After excluding all articles with no direct information relative to our subject and adding others found through other sources or identified through manual checking of references listed in the articles found, 60 articles were included in this review. Fig. 1 shows a PRISMA flow diagram summarizing the methodology, which was created following the indications of Page et al. (2021).

## 3 Purkinje cells in epilepsy

**Fig. 2:**
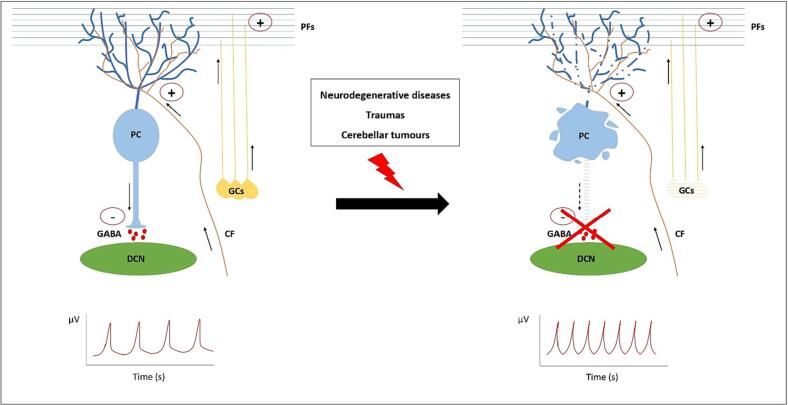
Role of Purkinje cells in epileptic seizures. The cerebellum is composed of different types of cells. These include granule cells and Purkinje cells (PCs) which communicate with each other. PCs are GABAergic neurons which exert an inhibitory effect on their targets, i.e., the deep cerebellar nuclei (DCN). They receive excitatory input from climbing fibres originating from the olivary nuclei and from parallel fibres, which are the axonal extensions of granule cells. In physiological conditions PCs integrate these excitatory signals and exert their inhibitory effect on the DCN. Conversely, in the presence of pathophysiological conditions such as cerebellar tumours, traumas, or neurodegenerative disease in which there is massive degeneration and loss of PCs, the function of these neurons fails. Since epilepsy can be traced to a loss of balance in neuronal communication, a state of hyperexcitability caused by loss of the inhibition from PCs is plausible. PC Purkinje cell. GCs granule cells. DCN deep cerebellar nuclei. PFs parallel fibres. CF climbing fibre.

In epilepsy, seizures are caused by disruption of the physiological balance between inhibitory and excitatory signals in the central nervous system (CNS) (Isaacson and Scanziani, 2011). Although the hippocampus has been described as the main seizure centre in the brain (Chatzikonstantinou, 2014), studies also show considerable cerebellar involvement in epilepsy (Streng and Krook-Magnuson, 2021; Ming et al., 2021). The cerebellum has an extensive network of outputs projecting from cerebellar nuclei to thalamic nuclei. The layers of PCs, which are GABAergic neurons, exert their inhibitory effect by releasing the neurotransmitter γ-aminobutyric acid (GABA), which acts on their targets, the deep cerebellar nuclei (DCN) (Roostaei et al., 2014). A central role for the cerebellum in epileptogenesis is supported by case reports and studies of animal models of cerebellar lesions associated with seizures (Ming et al., 2021). PCs provide the only inhibitory output of the cerebellum and are essential for cerebellar function. Accordingly, depletion of these cells disrupts the physiological balance between inhibitory and excitatory signals in the CNS. Generally, PCs have been found to be decreased in number in the cerebellum of patients with epilepsy versus controls (Rajjoub et al., 1976; Ming et al., 2021). Furthermore, microinjection of GABA agonists in mice may be correlated with the occurrence of epilepsy (Bittencourt et al., 2010), suggesting involvement of GABAergic neurons in epilepsy. Onset of seizures can be traced back to a state of hyperexcitability following loss of the inhibitory effect of PCs (Fig. 2) (Isaacson and Scanziani, 2011). Cerebellar stimulation results in improved seizure control and partial suppression of discharges (Davis et al., 1983; Chkhenkeli et al., 2004). Moreover, a mouse model of epilepsy showed a reduction in seizure duration after stimulation of PCs through optogenetic control (Krook-Magnuson et al., 2014). However, purky mice with extensive loss of PCs and an epilepsy phenotype showed a reduction in cell firing within cerebellar nuclei, probably due to a compensatory mechanism (Schwitalla et al., 2022). The mechanisms underlying seizure occurrence after PC loss are still unclear.

## 4 Progressive myoclonic epilepsy and Purkinje cells

The umbrella term PME covers a group of autosomal recessive disorders characterised by focal and generalised seizures, myoclonus, and progressive neurological dysfunction, including cerebellar ataxia and cognitive deterioration (Shahwan et al., 2005; Orsini et al., 2019). The main symptom is myoclonus, which tends to be multifocal affecting distal limb and face muscles primarily, and could be worsened by somatosensory reflexes (Faught, 2003; Shahwan et al., 2005). An electroencephalogram (EEG) showing an epileptic status with epileptiform discharges is a common feature of most PMEs (Acharya et al., 1995). Several diseases, such as Unverricht-Lundborg disease (*EPM1*), MERRF, NCLs, Gaucher disease, sialidosis, DRPLA and Lafora disease (*EPM2*) (Zupanc and Legros, 2004; Aguado et al., 2010; Knupp and Wirrell, 2014; Kälviäinen, 2015) lead to PME. Moreover, potassium channel mutations also result in PME, the mutated genes being *KCNC1*, encoding potassium voltage-gated channel subfamily C member 1, and *KCTD7*, which encodes for potassium channel tetramerization domain containing 7 (Van Bogaert, 2016; Park et al., 2019).

In the several diseases in which PME occurs, loss of the PC population can be attributed to different events. In NCLs, EPM2 and sialidosis, neurodegeneration is caused by gene deficiency with accumulation of intracellular inclusion bodies (Haltia, 2003; Verhalen et al., 2018; Khan and Sergi, 2018), whereas in EPM1-PME, it is caused by a loss of function of the cystatin B gene, which is an inhibitor of cysteine protease, therefore the resulting symptoms are attributed to excessive proteolysis (Turk and Bode, 1991).

Impairment or complete loss of PCs, leading to decline of cerebellar functions (Lehesjoki and Koskiniemi, 1998; Mascalchi et al., 2002; Fu et al., 2014), is a hallmark of many forms of PME. Loss of these neurons is described in some case reports and animal models of PMEs (Zhou et al., 1997; Kumada et al., 2000; Di Giaimo et al., 2002; Riccio et al., 2005; Ming et al., 2021). For instance, case reports of patients with PME associated with epileptic seizures and myoclonus related to a progressive deterioration of cerebellar cortex show massive loss of PCs and elevated protein glycation in this cell population (Nardelli et al., 1975; Hayashi, 2009).

Degeneration of PCs has also been described in some cases of EPM1 (Koskiniemi et al., 1974; Lehesjoki and Koskiniemi, 1998). The association of PME and EPM1 was evaluated using a mouse model with knockout of cystatin b, in which PME symptoms were associated with cerebellar cortical degeneration, particularly in the granule cell layer (Pennacchio et al., 1998). Although, this study found granule cell loss to be the main feature of neurodegeneration triggered by cystatin b knockout, a later study attributed this loss to PC damage, and the different PC phenotypes were explained by variable expression of cystatin b (Riccio et al., 2005). Moreover, a study in a mouse model of EPM1 (Shannon et al., 2002) showed patchy loss of PCs and Bergmann gliosis, indicating involvement of PCs in PME associated with cystatin b knockout. Instead, loss of PCs was not significant in animal models of EPM1.

In MERFF syndrome caused by a mitochondrial defect and characterised by PME, no significant loss of PCs was found, even though the percentage of mutant mtDNA was higher in these cells than in other brain cell subpopulations (Zhou et al., 1997).

Specific loss of GABAergic inhibitory cells and cerebellar interneurons has been described in myoclonus epilepsy and ataxia (MEAK), a form of PME caused by a mutation in *KCNC1* (Irie et al., 2014). PME has also been associated with mutations in another potassium channel gene, *KCTD7*, having been described in a family with three siblings showing symptoms of the disease. A gene expression study conducted to characterize the location of the protein found it to be massively expressed in PCs, suggesting that it has a key role in PC function (Farhan et al., 2014). Moreover, a progressive loss of PCs was observed in human patients with two novel *KCTD7* mutations and was consistent with the presence of myoclonic or generalized tonic-clonic seizures and abnormal EEGs (Dai et al., 2019). Similarly, myoclonic seizures and locomotor deficits correlated with loss of PCs were shown in a mouse model with a *Kctd7* deficiency (Liang et al., 2022).

## 5 Neuronal ceroid lipofuscinosis and Purkinje cells

The NCLs are rare genetic neurodegenerative disorders predominantly occurring during childhood and early adult life, with a worldwide prevalence ranging 2-4:1.000.000(Mole and Cotman, 2015; Simonati and Williams, 2022). Multiple studies over the past two decades have identified different forms of NCL associated with over 400 mutations in 13 genes (Mole and Cotman, 2015) (described in the literature and listed in the web-based NCL Mutation Database www.ucl.ac.uk/ncl/mutation) (Haltia and Goebel, 2013) mostly due to a missense mutation resulting in a loss-of-function. The NCL proteins have varying function and localisation: four types of NCL are caused by defects in lysosomal enzymes (CLN1, CLN2, CLN10, CLN13), two by defects in soluble lysosomal protein (CLN5, CLN11), and five by defects in lysosomal transmembrane proteins (CLN3, CLN4, CLN6, CLN7, CLN8) (Kyttälä et al., 2006; Jalanko and Braulke, 2009; Mukherjee et al., 2019). The CLN12 form of the disease is caused by variants in the *ATP13A2* gene, while mutation of a potassium channel gene causes CLN14 (Kollmann et al., 2013). As already mentioned, the NCLs are commonly considered a subgroup of the PMEs, with which they share certain characteristics; for example, as the disease progresses the EEG becomes flat and clinical epileptic activity worsens (Berkovic et al., 1993; Kravljanac et al., 2020). Although, myoclonic seizures are a common symptom of all the NCLs (Mole et al., 2020; Santavuori et al., 2000), mutations in *CLN4* and *CLN6* both cause the adult form of a PME syndrome called Kuf’s disease type A (Nita et al., 2016; Talbot et al., 2020). Kufs disease type A is difficult to diagnose and should be suspected in teenagers and young to middle-aged adults with PME (Berkovic et al., 2019). Although initially related only to PME (Kousi et al., 2012a; Van Bogaert et al., 2007), mutations in *KCTD7* have recently been related to CLN14 (Staropoli et al., 2012; Wang et al., 2022).

Neuronal loss in NCL is extensive and leads to CNS atrophy. Neurodegeneration is probably the result of different mechanisms, still to be clarified, such as impairment of lysosomal activity due to gene deficiency. Defects in neuronal lysosomal function also lead to dysregulation of autophagy and impairment of the intracellular degradation pathways essential for cell survival. A further feature common to storage diseases and PME syndrome is the presence of reactive glial cells (microglia and astrocytes), leading to increases in the same cytokines and chemokines (Sanz and Serratosa, 2020). Studies have shown that activation of these cells precedes or goes hand in hand with neuronal loss, and therefore serves as a marker for detecting neuronal impairment in NCLs (Francelle and Mazzulli, 2022; Takahashi et al., 2022). Neuronal loss is continuous and becomes more widespread as the disease progresses. Atrophy occurring much earlier in the cerebellum and thalamus than in other parts of the CNS has been described, accompanied by massive glial activation and accumulation of autofluorescent material (Kaminiów et al., 2022). Specifically, the cerebellar cells most affected by neurodegeneration are the granule cells and PCs, albeit with some temporospatial differences between the different NCLs. At autopsy CLN1 and CLN5 patients show a complete loss of PCs, and those with CLN4 show considerable loss, whereas patients with CLN3 display more pronounced loss of granule cells compared with PCs (Haltia, 2003).

### 5.1 CLN1

Mutations in *PPT1* (palmitoyl protein thioesterase 1) cause CLN1 disease, an INCL. PPT1 enzyme is localized in lysosomes, synaptic vesicles and axons (Hellsten et al., 1996; Lehtovirta et al., 2001; Ahtiainen et al., 2003). Deficient PPT1 activity resulted in neuronal cell loss in the CNS with pronounced PC and granule cell loss observed in the cerebellum of patients at autopsy (Haltia et al., 1973). A *PPT1*^-/-^ mouse model by Gupta et al. (2001) recapitulates some aspects of INCL disease, such as ataxia, spasticity and generalized myoclonus and seizures. Analysis of PPT1 expression in the wild-type mouse showed striking localization of the protein in PCs, hippocampus and thalamus. Autofluorescent storage material in PCs of PPT1 deficient mice highlights a pattern similar to the *PPT1 mRNA* expression. Moreover, histological analyses on mutant mice show a significant loss of PCs and gliosis compared to wild-type mice (Gupta et al., 2001). In subsequent research, loss of PCs in the *PPT1*^-/-^ mouse model was shown to begin at 3 months of age and become significant at 6 months. Reduction in the number of PCs is age-dependent and correlated with motor impairments, with EEG changes appearing at 6 months and onset of spontaneous seizures at 7 months (Griffey et al., 2006; Kielar et al., 2007). A comprehensive study on the onset and progression of forebrain pathology and seizures in *PPT1*^-/-^ mice, revealed that the loss of cortical and hippocampal interneurons is concomitant with seizures onset in *PPT1*^-/-^ mice (Kielar et al., 2007). Treatment of these *PPT1*^-/-^ mice via AAV2-mediated gene therapy appeared to reduce this seizure activity and prevent much of the underlying pathology (Macauley et al., 2009).

### 5.2 CLN2

CLN2 disease is associated with mutations in *TPP1* (the tripeptidyl peptidase 1 gene). Functional deficiency of the protein results in late infantile onset forms of NCL (Sleat et al., 1997; Vines and Warburton, 1998). In case reports of LINCL disease, autofluorescence material in the CNS and a progression to neurodegeneration are reported, with complete absence of PCs in the later stages (Goebel et al., 1999). Similarly, in a mouse model of CLN2 disease, PC loss was seen to increase with progression of the disease, with extensive Bergmann gliosis observed in the region affected by the neurodegeneration (Sleat et al., 2004; Chang et al., 2008). A *TPP1*^-/-^ mouse model did not show pronounced myoclonic epilepsy (Sleat et al., 2004), typical of CLN2 patients (Pérez-Poyato et al., 2013), brief seizures were observed only in the late-stage mice before mice died prematurely. However, a recent study of Takahashi et al. (2023) has defined the seizures phenotype of Cln2 knock-in mouse in more detail. In this study, log-term recording EEG revealed that 70% of these mice die within 3 minutes of their last seizure, suggesting a possible relationship between seizures and death. Moreover, histological analyses highlighted a more profound loss of cortical vs. hippocampal interneurons, suggesting that cortical pathology may be related to seizure etiology (Takahashi et al., 2023).

### 5.3 CLN3

The JNCL also known as Batten disease is the most common form of NCL. In 85% of cases, it is characterised by the so-called common deletion of 1kb in exons 7-8 of the *CLN3* gene (Mole, 2004). The typical clinical features of CLN3 disease are vision loss, motor and cognitive disorders, and seizures (Cotman and Lefrancois, 2021), and the condition leads to a reduced life expectancy with death in early adulthood. Batten disease is characterised primarily by impaired motor coordination with myoclonus and ataxia linked to brain atrophy (Nardocci et al., 1995), shown on magnetic resonance imaging (Autti et al., 1996). A mouse model of *Cln3*^-/-^ showed a motor deficit related to a cerebellar dysfunction with granule cell layer loss due to a glutamatergic dysregulation (Kovács et al., 2006). Subsequently, in the same model, extensive Bergmann gliosis and PC loss led the authors to suggest that the granule cell layer dysfunction is probably a secondary consequence. Pronounced PC loss and glial activation were also correlated with a more aggressive and rapid disease course in CLN3 patients, compared with slower progression in the presence of less significant PC loss (Weimer et al., 2009). Loss of GABAergic interneurons was described in a knock-out mouse model of CLN3 disease specifically in hippocampal region, became evident with increased age (Pontikis et al., 2004). Another *Cln3* knockout mouse model displayed pharmacologically-induced seizures and showed preferential loss of GABAergic neurons through an autoimmune response (Chattopadhyay et al., 2002; Ramachandran et al., 2009). Immunostaining in a *Cln3*^Δex7/8^ knock-in mouse model revealed deposits, before birth in different regions including hippocampus, thalamus, and PCs in the cerebellum (Cotman et al., 2002). Although, this knock-in model display motor impairment, neurodevelopment delay and decrease survival, common symptoms of JNCL including spontaneous seizures and visual loss were not displayed (Cotman et al., 2002; Osório et al., 2009). Epilepsy has been described in patients with CLN3 disease (Arntsen et al., 2019; Abdennadher et al., 2021) and *CLN3* has been mapped as an “epilepsy gene” (Delgado-Escueta et al., 1994). In a zebrafish model, EEGs showed high-amplitude spiking similar to what is seen in humans; moreover, the authors observed a lack of axonal organisation in the brain, characterised in particular by loss of axons in the optic tectum and developing cerebellum, and particularly marked loss of neurons in the midbrain, hindbrain, retina and cerebellum, but a specific analysis of the PC cell layer was not performed (Wager et al., 2016).

### 5.4 CLN4

CLN4 disease is associated with mutations in *DNAJC5* encoding a cytosolic vesicle-associated co-chaperone CSP-α (Lee et al., 2023; Zhang et al., 2013). CLN4, also known as Parry or Kufs disease with adult onset, is the only autosomal dominant type of NCL (Mole and Cotman, 2015; Schulz et al., 2013). Clinical symptoms include ataxia, seizures, and progressive myoclonus without ocular involvement (Kousi et al., 2012b). Age at onset of CLN4 ranges from 25 to 40 years. All patients show epilepsy and myoclonus, as either the first or later symptoms (Naseri et al., 2021). Cerebellar atrophy is reported in most of the cases described to date (Jarrett et al., 2018; Nijssen et al., 2003; Josephson et al., 2001; Burneo et al., 2003). Involvement of PCs is described in different studies, as patchy loss of PCs (Josephson et al., 2001), and the presence of storage material in PCs in patients with epilepsy (Sadzot et al., 2000). Moreover, CSP-α is essential to prevent neurodegeneration in cultures of GABAergic hippocampal neurons compare to glutamatergic terminals (García-Junco-Clemente et al., 2010).

### 5.5 CLN5

Mutations in *CLN5* have been shown to cause a variant form of LINCL, with visual impairment, myoclonus and epilepsy, and early involvement of the cerebellum (Cannelli et al., 2007). CLN5 is a soluble lysosomal glycoprotein, and its mutations are associated with lysosomal dysfunction (Jules et al., 2017; Basak et al., 2021). In a study from Tyynelä et al. (1997) was shown a cerebellar atrophy with a massive loss of both PCs and granule cells in 3 patients with vLINCL (Tyynelä et al., 1997). Moreover, studies on *CLN5* expression in humans and in mice showed strong *CLN5* expression in the PC layer (Heinonen et al., 2000; Holmberg et al., 2004).Six-month-old Cln5-/- mice show a selective loss of GABAergic neurons the cerebral cortex, hippocampus, thalamic nuclei, midbrain and cerebellum. However, progressive motor abnormalities or development of spontaneous seizures were not detected in this model (Kopra et al., 2004) . Thus, the possibility of a role for PCs in the onset and progression of disease related to CLN5 deficiency needs to be further evaluated in future studies.

### 5.6 CLN6

Mutations in *CLN6* cause both LINCL and ANCL forms (Mole and Cotman, 2015). *CLN6* encodes a membrane protein in the endoplasmic reticulum which has been suggested to be involved in autophagy and biometal homeostasis (Mole et al., 2020). More recently, Kanninen et al. (2013) showed a strict correlation between CLN6 expression and a cellular biometal transporter protein, Zip7. In CLN6 mouse model brains, expression of Zip7 in PCs was altered, being shown to be higher than in control brains. Seizures are a typical symptom of CLN6 disease (Canafoglia et al., 2015; Berkovic et al., 2019), especially in the adult form (Rus et al., 2022), in which patchy loss of PCs has been observed (Robertson et al., 2008). However, in the abovementioned mouse model, only PC involvement in motor symptoms was evaluated; the possible involvement of these cells in epileptic status in this model thus needs to be further evaluated. Alternatively, it has been hypothesized that in the brains of South Hampshire sheep with CLN6 disease, seizures are attributable to the profound loss of parvalbumin immunoreactive interneurons in these sheep (Oswald et al., 2008).

### 5.7 CLN7

CLN7 disease is caused by a bi-allelic mutation in *MFSD8* (major facilitator superfamily domain containing 8), which encodes a transmembrane lysosomal transporter with unknown function (Siintola et al., 2007) and is associated with a vLINCL form (Mole and Cotman, 2015). CLN7 disease progresses rapidly from onset, with ataxia, visual impairment and myoclonus epilepsy culminating in premature death (Kousi et al., 2009). In rat brain the lysosomal transporter has been found to be expressed in the CNS, especially the cerebellum and hippocampus (Sharifi et al., 2010). In a mouse model of CLN7 disease with a disruption of *Mfsd8* gene, through a gene-trap cassette predicted to truncate Cln7 protein, a strong accumulation of autofluorescent material has been observed in PCs, related to the highest expression of *MSFD8* in the PC layer. Furthermore, in PCs, severe immunoreactivity to the accumulation of SCMAS (subunit c of mitochondrial ATP synthase), which is the main hallmark of CLN7 disease progression, has been shown (Damme et al., 2014; Brandenstein et al., 2016). Additionally, *Mfsd8*-knockout mouse model of CLN7 developed by Brandenstein et al. (2016) displayed myoclonus epilepsy with inability to survive beyond 10-11 months of age.

### 5.8 CLN8

Mutations in the gene *CLN8* cause an vLINCL variant characterised by early onset and rapid progression (Striano et al., 2007). Patients with CLN8 disease have shown drug-resistant epilepsy (Ranta et al., 1999; Katata et al., 2016). CLN8 is a transmembrane protein that localises to the ER and the ER-Golgi intermediate compartment in neuronal and non-neuronal cells (Kollmann et al., 2013). *Cln8* has been found to be extensively expressed in developed mouse organs, especially in adult cerebellum, and this *Cln8* expression was reported to be 156% of expression in the cortex (Lonka et al., 2005). In the kindling model of experimental epilepsy, a rapid up-regulation of *Cln8* was observed in the hippocampus, suggesting a possible neuroprotective role of the gene (Lonka et al., 2005). Although cerebellar atrophy has been described in CLN8 patients, the role of PCs in the disease onset and progression needs further investigation. Nonetheless, CLN8 was found to be highly expressed in PCs (Passantino et al., 2013), and in a mixed breed dog affected by CLN8 disease histopathology marked accumulation of storage material was found in these cells (Guo et al., 2014). CLN8 is closely involved in the ceramide biosynthesis pathway due to its role in lipid metabolism (Haddad et al., 2012). Consequently, in a mutant mouse with impaired ceramide biosynthesis, PCs were the most affected cells from lipofuscin accumulation (Zhao et al., 2011), suggesting a role for PCs in CLN8 disease onset.

### 5.9 CLN10

CLN10 refers to types of congenital NCL, in which clinical manifestations are already present at birth (Schulz et al., 2013). Postnatal epileptic seizures and congenital microcephaly lead to premature death within hours or weeks of birth (Kousi et al., 2012b). CLN10 disease may also manifest as late-infantile, juvenile and adult forms of NCL (Schulz et al., 2013), and it is linked to mutations in the *CTSD* gene which encodes a cathepsin D lysosomal enzyme (Siintola et al., 2006).

In case reports of congenital CLN10 disease, the cerebellum has been described as reduced in size and showing marked atrophy (Varvagiannis et al., 2018; Meyer et al., 2015). Moreover, severe myoclonic seizures were accompanied by lipopigments and extensive loss of neurons in the cerebellar region (Meyer et al., 2015). A knock-out mouse model of Cathepsin D manifests seizures at P20 and died premature death (Koike et al., 2000). Electrophysiological analysis on hippocampal slices, from knock-out mouse model, exhibit spontaneous burst discharges at late-symptomatic stage. Actually, no synaptic alterations were observed in hippocampus of mice without seizures in earlier stages (P16), indicating neurodegeneration precede seizures onset (Koch et al., 2011). A subsequent study on a mouse model with CTSD deficiency only in PCs shows massive presence of granular osmiophilic deposits, strong immunoreactivity for LAMP-1 (lysosomal-associated membrane protein), and reduction of PCs but no electrophysiological analysis was performed (Koike et al., 2017).

### 5.10 CLN11

Mutation of the *GRN* gene has been associated with CLN11, a form of NCL in which there is a progranulin deficiency (Smith et al., 2012). Patients with progranulin deficiency showed vision loss, seizures, and cognitive and language deterioration (Smith et al., 2012; Huin et al., 2020). Associations between progranulin deficiency and NCL were found in a mouse model with a *Grn*^*-/-*^ genotype, which showed accumulation of autofluorescent lipopigments in neurons and retinas (Smith et al., 2012; Hafler et al., 2014). To date, the literature contains no strong evidence of PC involvement in CLN11 disease, although high expression of *progranulin* mRNA was found in specific populations of neurons, including granule cells of hippocampus and PCs (Daniel et al., 2000) and *PGRN* mRNA is constantly expressed in PCs after 50 weeks, compared to the downregulation observed in other brain regions and cell subpopulations (Matsuwaki et al., 2011). Conversely, *CLN11* knockout mice showed differences in PCs maturation with higher density of PCs dendrites, a finding which may have a bearing on the CLN11 disease phenotype (Matsuwaki et al., 2015).

### 5.11 CLN12

In a family with typical NCL pathology, mutation of the parkinsonism gene *ATP13A2* was described as correlated with a JNCL form that the authors proposed should be termed CLN12 (Bras et al., 2012). Mutation in *ATP13A2* was associated with the presence of lipofuscin deposits in cortex, cerebellum, retina and basal nuclei (Bras et al., 2012). ATP13A2 has been shown to localise to the membranes of endosomes and lysosomes and to multivescicular bodies (Kong et al., 2014). ATP13A2 is predicted to act as an active transporter of inorganic cations, in fact its deficiency impairs lysosomal function (Dehay et al., 2012). Epilepsy was not found to be prominent in patients with CLN12, and involvement of PCs, shown by elevated autofluorescent storage bodies, has been described only in Australian cattle dogs found to have a missense mutation in *ATP13A2* (Schmutz et al., 2019).

### 5.12 CLN13

CLN13 disease was originally described in a cathepsin F knockout mouse model that showed an accumulation of autofluorescent granules in the cerebral cortex, hypothalamus and cerebellar PCs, and two mice developed seizures (Tang et al., 2006). Later, the CLN13 form was studied in families with Kufs disease type B and mutations in *CTSF* were linked to an ANCL form (Smith et al., 2013). CLN13 has not been directly associated with PME phenotypes, having been found to be associated mainly with ataxia and dementia (Minassian, 2014).

### 5.13 CLN14

As described above, mutations in *KCTD7* have been commonly linked to various forms of PME. In a study by Staropoli et al. (2012), mutation in KCTD7 protein was shown to be associated with a form of NCL called CLN14. In the mouse brain, *KCTD7* has been found to be highly expressed in the hippocampus, cerebral cortex, and PCs. A *Kctd7*-deficient mice display robust epileptiform activity and locomotor deficits with elevated expression of *Kctd7* preferentially in PCs and hippocampal neurons. Additionally, histological analysis of a *Kctd7* knockout mouse model showed progressive loss of PCs and Bergmann gliosis, suggesting that *KCTD7* has an important role in the long-term survival of PCs (Liang et al., 2022).

## 6 Discussion

Degeneration and death of PCs, which provide the only inhibitory output of the cerebellum, is described in many neurodegenerative diseases. PC involvement has been studied in ataxia, HD, AD, PD and other forms of neurodegeneration. In diseases such as cerebellar ataxia, motor dysfunctions have been associated with cerebellar atrophy, most evident at the level of the PCs (Cook et al., 2021). Although the cerebellum is commonly associated with motor coordination, studies have demonstrated its role in cognitive functions (Carta et al., 2019), too, as shown by evidence collected in various pathological conditions: AD, PD and PMEs (Soria Lopez et al., 2019; Aarsland et al., 2017; Satishchandra and Sinha, 2010). For instance, both decreased PC excitability and loss of PCs have been found in patients with autism spectrum disorders showing impairment of social behaviour (Rogers et al., 2013). Further evidence of the importance of PCs in cognitive function has been found in a mouse model of AD, which showed massive reduction of the number of PCs compared with cells in other regions of the CNS (Mavroudis et al., 2019).

As described by Krook-Magnuson et al. (2014), loss of the direct inhibition of the hippocampal region by PCs possibly leads to hyperexcitability resulting in epileptic seizures. However, the findings of the few studies exploring the direct role played by PCs in epilepsy are controversial, due to disease and patient variability. That said, the involvement of PCs in other genetic forms of myoclonic epilepsy, such as severe myoclonic epilepsy in infancy (SMEI), has been validated. A mouse model of SMEI lacking the Na(V)1.1 channel showed reduced electrical excitability of PCs. Immunohistochemical study demonstrated that Na(V)1.1 is the primary sodium channel isoform expressed in PCs, and thus that Na(V)1.1 is a regulator of GABAergic PC activation (Kalume et al., 2007). Accordingly, epileptic seizures in PME can be linked to loss of the physiological balance between inhibitory and excitatory signals in the CNS caused by degeneration of inhibitory neurons including PCs, which are directly and indirectly responsible for regulating hippocampus excitability. It must be said that the role of other GABAergic neurons in epilepsy onset in NCLs was already described. Pronounced loss of GABAergic interneurons was evidenced in human and animal models of NCLs (Cooper et al., 1999; Bronson et al., 1998; Koike et al., 2000; Cooper et al., 2002; Cotman et al., 2002; Kopra et al., 2004; Sharifi et al., 2010; García-Junco-Clemente et al., 2010; Takahashi et al., 2023). To date, a severe loss of hippocampal interneurons was described in CLN1, CLN2, CLN5 and CLN8 (Tyynelä et al., 2004). Indeed, a massive loss of GABAergic neurons in lysosomal storage disease is mainly due to a high metabolic rate, therefore, these cells are more susceptible to metabolic insult caused by traffic/autophagy impairment (Walkley and March, 1993). Evidence suggests the involvement of PCs in many forms of PME, such as EPM1, MERFF disease and lysosomal storage diseases. The present review highlights how PC loss may contributes to deterioration of motor function, epilepsy onset and cognitive decline, both in humans and in animal models of different NCLs. Not all the forms of NCL are classical PMEs, but most display epilepsy or myoclonic epilepsy at some point. The key role of PCs in NCLs has been demonstrated through examination of cerebellar pathogenesis in which loss of these cells is prominent. Table 1 summarizes Purkinje cells phenotype, involvement of other inhibitory neurons and seizures in different forms of NCLs. Moreover, most of the genes associated with NCLs have been found to be highly expressed in PCs, suggesting a critical role of these cells in disease onset. A role of PCs in seizures and epileptic status has been described in patients with chronic epilepsy who showed cerebellar atrophy, involving the PCs in particular. Furthermore, a correlation between PC loss and increased duration of seizures was found in some patients (Crooks et al., 2000). However, most studies on epilepsy focus on the status of “hyperexcitability” in the cerebral cortex and fail to look at the role of the cerebellar cortex and the question of how the cerebellar atrophy seen in most epilepsy disorders may partly underlie seizure onset. Finally, not all animal models of NCL display an epileptic status, signature of the human forms, even if most of the mouse models display at least a degree in the number of PCs, this suggests a possible threshold of PCs loss. However, this might be due to the need of other cell populations involvement in seizure onset, or to the variability of the phenotypes associated with the different types of mutation in *CLN* genes, also observed in humans. Moreover, loss of GABAergic inhibitory interneurons was observed mostly in non-murine models (March et al., 1995; Oswald et al., 2001). Suggesting that murine models are probably not suitable for highlighting the correlation between loss of inhibitory neurons and the onset of seizures. Nevertheless, the precise mechanism by which PCs attenuate epilepsy through their inhibitory effect on the neurons of cerebral cortex remains to be fully elucidated.

**Table 1:** NCL subtypes, Purkinje cell changes, involvement of other inhibitory neurons in humans and animals and epilepsy observed in animal models.

Subtypes of NCL	Phenotype of Purkinje cells in patients	Involvement of other inhibitory neurons	Phenotype of Purkinje cells in animal models	Seizures in animal models
*CLN1/PPT1*	Reduction in number at autopsy	Loss of thalamocortical interneurons in mouse model	Reduction in number in mouse	Spontaneous seizures at 7 mo
*CLN2/TPP1*	Complete loss at final stages	Reduction in cortical neurons in mouse with seizures	Reduction paired with Bergman gliosis in mouse	Progressive in nature and dead-seizures hypothesis
*CLN3*	Extensive loss in more severe patients	Loss of GABAergic neurons in hippocampus.	Drop of GABAergic neurons and Bergmann gliosis in mice. Axonal disorganization in cerebellum in Zebrafish	Alteration in EEGs in Zebrafish
*CLN4/DNAJC5*	Diffuse loss, massive presence of autofluorescent storage material and alteration in dendrities architecture	Preferential neurodegeneration of hippocampal GABAergic neurons.	No evidence	No evidence
*CLN5*	Elevated expression of the gene in neurons	Loss of GABAergic neurons in hippocampus, thalamus and cerebral cortex.	Loss of GABAergic neurons in cerebellum	No evidence
*CLN6*	Slight loss	Neurodegeneration of parvalbumin immunoreactive interneurons	Evidence only on expression of the gene in the cells	No evidence
*CLN7/MFSD8*	Strong accumulation of autofluorescent material. Loss in later stages	Elevated vulnerability in CA2 hippocampal sector	Extensive accumulation of SCMAS and autofluorescent storage material in mouse	Disabling and fatal epilepsy
*CLN8*	Elevated expression of the gene specifically in the cerebellum	Up-regulation of *Cln8* expression in kindling model of experimental epilepsy	Strong accumulation of autofluorescent storage material in a dog. Neuronal degeneration in a mouse with impaired ceramide byosinthesis.	No evidence
*CLN10/CTSD*	Reduction in size with degeneration and accumulation of autofluorescent storage material	Hippocampal degeneration at later stages in mouse model	Accumulation of storage material, immunoreactivity for lysosomal markers and neuronal degeneration in mice.	Burst discharge at later stage
*CLN11/GRN*	Specific expression of *grn mRNA* in neurons	Specific expression of *grn* in hippocampal neurons	Accumulation of autofluorescent storage material and incorrect neuronal maturation in mouse model.	No evidence
*CLN12/ATP13A2*	No evidence	No evidence	Strong accumulation of autofluorescent storage material in dogs.	No evidence
*CLN13/CTSF*	Not directly associated with PMEs	No evidence	Not directly associated with PMEs	Not directly associated with PMEs
*CLN14/KCTD7*	Progressive loss associated with EEG alterations	Elevated levels of *mRNA* and protein of *kctd7* in hippocampal neurons	Loss and neuronal degeneration with Bergmann gliosis in mouse.	Myoclonic seizures

## 7 Conclusion

This analysis of the literature revealed evidence of the important contribution played by the Purkinje cells in the phenotype of different forms of neuronal ceroid lipofuscinosis and other progressive myoclonic epilepsies, highlighting the association between loss of inhibitory cells, including Purkinje cells and seizure onset. Future studies must improve the knowledge in how the loss of PCs could contribute, together with other GABAergic neurons to seizures onset and progression,using suitable animal models to characterize seizure phenotypes . Moreover, the evidence collected underlines the need to consider the Purkinje cells as a target of potential therapies aimed at modifying/delaying the disease progression by preventing their death or stimulating their GABAergic function.

## Author Contributions

SB, and MM: conceptualization. SB: methodology. SB: investigation. SB: resources and writing original draft preparation. MM and SB: writing review and editing. FG: visualization. MM: supervision and funding acquisition. MM: project administration.

## Declaration of Competing Interest

The authors declare that the research was conducted in the absence of any commercial or financial relationships that could be construed as a potential conflict of interest.

## Data Availability

No data was used for the research described in the article.
